# The Use of Insecticide against Lice

**Published:** 1918-01

**Authors:** 


***The Use of Insecticide Against Lice.*** By A. BACOT, Entomologist, Lister Institute. Abs. from the *Brit. Med. Jour.*, Sept. 30, 1916.

B. presents the results of experiments to determine the value of various insecticides and the best methods of employing them.

The following preparations were tested by suspending small bags of lice in close proximity to bags impregnated with the substance to be tested : Naphthaline, Sulphur, Cresylic acid, Iodoform, Vermijelli, a solution of naphthaline, kerosene oil, and benzol, a mixture of vermijelli and cresylic acid, cytisine, a mixture of vermijelli with phenol, and one of iodoform, cytisine, and sulphur. Care was taken that the condition of the lice, especially their feeding, should be normal. The tests were made at various points on the body with the usual clothing conditions.

B. concludes that the diffusion of all of these preparations except naphthaline is too slight for them to serve as anything but contact remedies. Impregnation of underclothing would be the only method of making the use of iodoform or cytisine effective; but the cost of this procedure is prohibitive. Naphthaline used as a powder or in sachets is practically of small value. Used as a liquid for impregnating clothing it is an ideal remedy, for it acts quickly and surely; but it loses its power the second day. Nothing is gained by adding iodoform to it.

A series of experiments was also undertaken to show the effect upon breeding produced by impregnation with diluted, low cost solutions. The author objects to the use of cytisine for impregnation not merely because of its cost, but also because of its probable toxic effect upon the skin if used in sufficient strength to affect the lice. He believes, however, that the cheapest, most effective, and most lasting preparation for impregnating the clothing, and one which does not irritate the skin, is a mixture of crude liquid carbolic acid and soft soap. " The emulsion ", he states, " should consist of 45 o/o to 50 o/o of soft soap, combined by heating with 50 o/o to 55 o/o of the crude carbolic. The strength of the solution to impregnate the garments should be 5 o/o of the emulsion in warm water, 3 o/o being too weak for practical use; while solutions above 5 o/o might irritate the skin.... The cost, apart from labor, is about one halfpenny a shirt[1]. "

B. thinks that some sensitive skins may be irritated by the small percentage of cresols in the shirts, and urges practical experimentation on a large scale to determine the seriousness of this risk and also the practical effectiveness of the remedy in ridding the troops of lice.

1. B. also tried rosin soap in a crude carbolic acid emulsion allied to the Isthmian mosquito lavicide used by the Panama authorities. He found, however, that this did not give a perfect emulsion, and that it was unreliable for impregnating flannel. This was also true of emulsions of soft soap and crude carbolic acid in which less than 40 per cent of soft soap was used.

